# Fabry disease Schwann cells release p11 to induce sensory neuron hyperactivity

**DOI:** 10.1172/jci.insight.172869

**Published:** 2024-04-22

**Authors:** Tyler B. Waltz, Dongman Chao, Eve K. Prodoehl, Jonathan D. Enders, Vanessa L. Ehlers, Bhavya S. Dharanikota, Nancy M. Dahms, Elena Isaeva, Quinn H. Hogan, Bin Pan, Cheryl L. Stucky

**Affiliations:** 1 Department of Cell Biology, Neurobiology & Anatomy;; 2 Department of Anesthesiology; and; 3 Department of Biochemistry, Medical College of Wisconsin, Milwaukee, Wisconsin, USA.

**Keywords:** Neuroscience, Ion channels, Neurological disorders, Pain

## Abstract

Patients with Fabry disease suffer from chronic debilitating pain and peripheral sensory neuropathy with minimal treatment options, but the cellular drivers of this pain are unknown. Here, we propose a mechanism we believe to be novel in which altered signaling between Schwann cells and sensory neurons underlies the peripheral sensory nerve dysfunction we observed in a genetic rat model of Fabry disease. Using in vivo and in vitro electrophysiological recordings, we demonstrated that Fabry rat sensory neurons exhibited pronounced hyperexcitability. Schwann cells probably contributed to this finding because application of mediators released from cultured Fabry Schwann cells induced spontaneous activity and hyperexcitability in naive sensory neurons. We examined putative algogenic mediators using proteomic analysis and found that Fabry Schwann cells released elevated levels of the protein p11 (S100A10), which induced sensory neuron hyperexcitability. Removal of p11 from Fabry Schwann cell media caused hyperpolarization of neuronal resting membrane potentials, indicating that p11 may contribute to the excessive neuronal excitability caused by Fabry Schwann cells. These findings demonstrate that sensory neurons from rats with Fabry disease exhibit hyperactivity caused in part by Schwann cell release of the protein p11.

## Abstract 2

Chronic debilitating pain in Fabry disease is driven by Schwann cell release of p11, which causes sensory neuron hyperexcitability.

## Introduction

Fabry disease, the most common X-linked lysosomal storage disease, is caused by a deficiency in the enzyme alpha-galactosidase A (α-Gal A), which results in chronic intracellular accumulation of glycosphingolipids in multiple tissues, including sensory dorsal root ganglion (DRG) neurons. Though initially considered a rare genetic disorder, recent studies suggest Fabry disease affects up to 1:1,600 individuals (1, 2) and is underdiagnosed (3). The most debilitating complication is childhood onset of severe chronic pain, described as mechanical allodynia and burning in the hands and feet. Many patients also endure recurring attacks of intense episodic pain that occur spontaneously or are triggered by extreme temperature, fever, fatigue, or stress (4). Existing treatments for pain are inadequate, and enzyme replacement therapy does not alleviate the pain (5–8). The pathophysiological mechanisms underlying the chronic and episodic pain in Fabry disease are poorly understood, yet this topic receives little research attention. Fabry pain has neuropathic attributes, and patients display evidence of peripheral neuropathy, including structural abnormalities in the DRG and peripheral nerves (9–14). However, it has not been established that pain in Fabry disease is specifically attributable to sensory neuron dysfunction, as pain may be attributed to central structures, including the spinal cord and brain (15–17). Furthermore, peripheral nerves from patients with Fabry disease show morphological changes in Schwann cells (11, 13, 18). Emerging evidence suggests that Schwann cells, which are critical for maintaining healthy nerve function, can induce pain by directly increasing the activity of nociceptive neurons (19–21), though it is unclear how Schwann cells elicit these effects. Here, we used a recently characterized genetic rat model (22) to investigate how peripheral sensory neurons and Schwann cells contribute to Fabry disease pain.

## Results

### Peripheral sensory neurons from Fabry rats exhibit spontaneous activity and mechanical sensitization.

**Figure 1:**
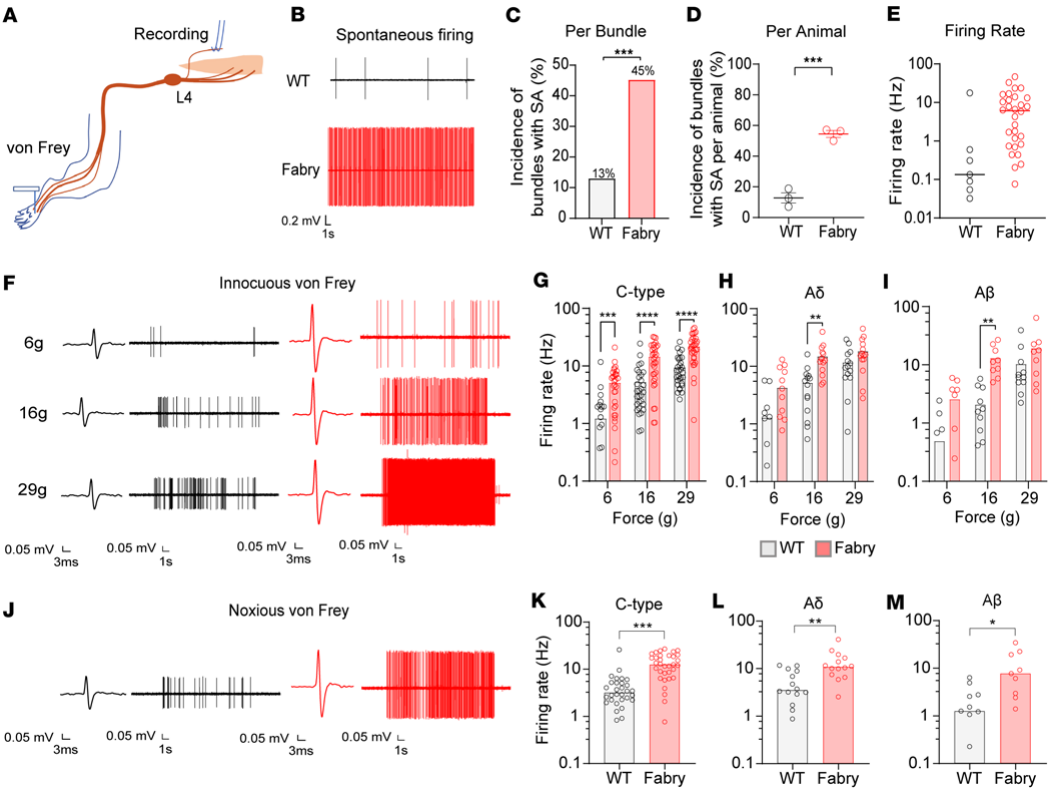
Peripheral dorsal roots from Fabry rats exhibit spontaneous activity and mechanical hypersensitivity. (**A**) Lumbar 4 (L4) dorsal roots from Fabry or WT rats were teased into bundles and recorded without external stimulation or with von Frey stimulation of the hind paw. (**B**) Representative traces of spontaneously firing dorsal roots from Fabry or WT rats. (**C**) Teased bundles from Fabry rats exhibited a higher proportion of spontaneous activity as analyzed per teased bundle or (**D**) per animal. (**E**) Spontaneous firing frequency between Fabry (red) and WT (black) rats (unpaired Student’s *t* test, *P* = 0.16). (**F**) Representative traces of recorded teased single-unit C-fiber activity due to innocuous von Frey stimulation of the plantar hind paw. Single (**G**) C-, (**H**) Aδ-, and (**I**) Aβ-units from Fabry rats exhibit increased firing frequency due to graded innocuous von Frey stimulation. (**J**) Representative traces of C-fiber activity due to stimulation of the hind paw with a modified von Frey filament; filament was modified with a tungsten tip to be noxious. (**K**) C-, (**L**) Aδ-, and (**M**) Aβ-units from Fabry rats exhibit increased firing frequency due to noxious von Frey stimulation of the hind paw. Fabry = red, WT = black. *n* = 3 rats per genotype. Data for (**C** and **G**–**I**) reported as mean, (**D**) as mean ± SEM, (**E** and **K**–**M**) as median. (**C** and **K**–**M**) Unpaired Student’s *t* test, (**G**–**I**) 2-way ANOVA significant main effect of treatment, Bonferroni post hoc comparison. * *P* < 0.05, ** *P* < 0.01, *** *P* < 0.001, **** *P* < 0.0001. SA, spontaneous activity.

Rats with completely deficient α-Gal A activity (Fabry rats) (22) exhibit ongoing, spontaneous pain behaviors (23). We first determined whether this is attributable to spontaneous activity in peripheral sensory neurons, as is commonly the case in other preclinical neuropathic pain models and human patients (24–34). Specifically, we used in vivo dorsal root teased-fiber electrophysiological recordings in anesthetized rats to determine whether peripheral nerves in Fabry rats exhibit spontaneous activity (Figure 1A). L4 dorsal roots of Fabry and WT rats were teased into fine bundles, and the incidence and frequency of spontaneous action potentials were recorded (Figure 1B). Fabry rats displayed a higher proportion of spontaneous afferent activity per bundle (Figure 1C) and per animal (Figure 1D) compared with WT rats. Dorsal root bundles from Fabry rats also exhibited a trend toward increased spontaneous firing frequency (Figure 1E).

Patients with Fabry disease often experience mechanical allodynia in the hands and feet, and Fabry rodent models exhibit behavioral hypersensitivity to mechanical stimulation of the hind paw (4, 13, 22). Therefore, we next asked whether peripheral sensory nerves in Fabry rats exhibit altered responses to innocuous or noxious mechanical stimuli applied to the hind paw. Single units recorded in dorsal roots from anesthetized Fabry and WT rats were classified as C-, Aδ-, or Aβ-fibers based on conduction velocity as measured via electrical stimulation of the sciatic nerve. A graded, innocuous von Frey stimulation (6, 16, 29 g) was applied to the plantar foot and unit response was recorded. Aβ-, Aδ-, and C-fibers from Fabry rats exhibited increased firing frequency in response to innocuous (Figure 1, F–I) and noxious mechanical stimulation (modified von Frey filament with a finely pointed tungsten tip) of the hind paw (Figure 1, J–M). There were no differences in the median conduction velocity of all fibers between Fabry and WT rats (Supplemental Figure 1A; supplemental material available online with this article; https://doi.org/10.1172/jci.insight.172869DS1). These data indicate that sensory neurons in Fabry rats exhibit spontaneous activity and enhanced responses to mechanical stimuli, consistent with a large- and small-fiber sensory neuropathy.

One potential source of spontaneous activity is the regenerative processes initiated in the growth cone of injured axons during their regrowth (35). We previously demonstrated decreased myelinated and unmyelinated fiber density in sensory nerves compared with controls, as well as glycosphingolipid accumulation within axons (36). Additionally, Fabry DRGs displayed a similar number of neuronal somata and an absence of satellite glial cell proliferation that followed degeneration of somata (37) (Supplemental Figure 2, A and B). Together, these findings suggest that glycosphingolipid accumulation in Fabry disease causes denervation, resulting in spontaneous neuronal activity (38–40) and potentially contributing to pain behaviors.

### Small-diameter sensory neurons from Fabry rats exhibit spontaneous activity and hyperexcitability.

**Figure 2:**
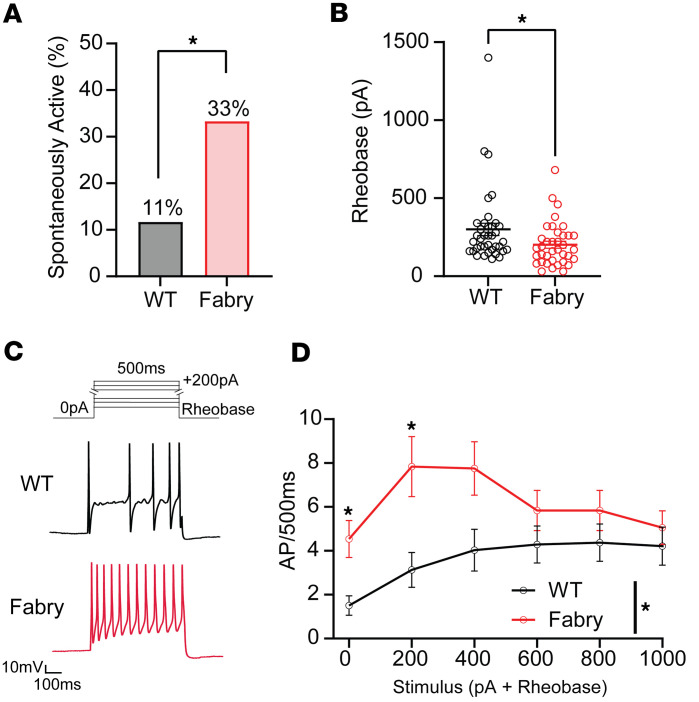
Isolated small-diameter DRG neurons isolated from Fabry rats exhibit spontaneous activity and current-evoked hyperexcitability. (**A**) A higher proportion of isolated Fabry DRG neuron somata exhibit spontaneous activity compared with WT. (**B**) Minimal action potential current threshold (rheobase) of Fabry neurons is reduced compared with WT neurons. (**C**) Current protocol for determining current-evoked firing frequency of Fabry and WT neurons with representative traces of Fabry and WT neurons undergoing current stimulation of 200 pA above rheobase for 500 ms. (**D**) Fabry DRG neurons exhibit increased firing frequency due to suprathreshold current stimulation. (**A**) *n* = 33–34 neurons from 6 animals per genotype; (**B**–**D**) *n* = 37–38 neurons from 8 animals per genotype. Data for **A** reported as mean, **B** and **D** as mean ± SEM. (**A**) χ^2^ Fisher’s exact post hoc comparison, (**B**) unpaired Student’s *t* test, (**D**) 2-way repeated measures ANOVA significant main effect of treatment, Bonferroni post hoc comparison. * *P* < 0.05. AP, action potential.

The sensory neuron soma is the established model for assessing excitability of the sensory neuron membrane. Accordingly, we examined excitability of dissociated sensory neuron somata from Fabry rats. DRG somata from Fabry rats retain glycosphingolipid accumulation after dissociation (22). In contrast with our previous patch clamp electrophysiology studies on Fabry DRG neurons (22), we compared neurons of similar diameter per genotype in this study (Supplemental Table 1), as cell size and capacitance influence neuron excitability (41). A higher proportion of small-diameter DRG neurons from Fabry rats displayed spontaneous activity compared with WT controls (Figure 2A). Fabry neuronal somata exhibited reduced action potential current thresholds, or rheobase (Figure 2B). Fabry neurons also showed increased firing frequencies evoked by suprathreshold depolarizing current injections compared with WT somata (Figure 2, C and D). Other passive and current-evoked membrane properties recorded from small-diameter DRG neurons were not different between genotypes (Supplemental Table 1). Neuronal somata larger than 32 µm exhibited a trend for enhanced firing frequency elicited by stimuli from 600 to 1,600 rheobase + pA, though the trend was not statistically significant (Supplemental Figure 3 and Supplemental Table 2). Although dissociation may alter the functional properties of the soma, our observations support the possibility that hyperactivity of small-diameter nociceptor somata may contribute to the spontaneous activity of single units recorded in the dorsal root and pain behaviors observed in Fabry rats.

### Schwann cells in Fabry rats exhibit abnormal morphology.

**Figure 3:**
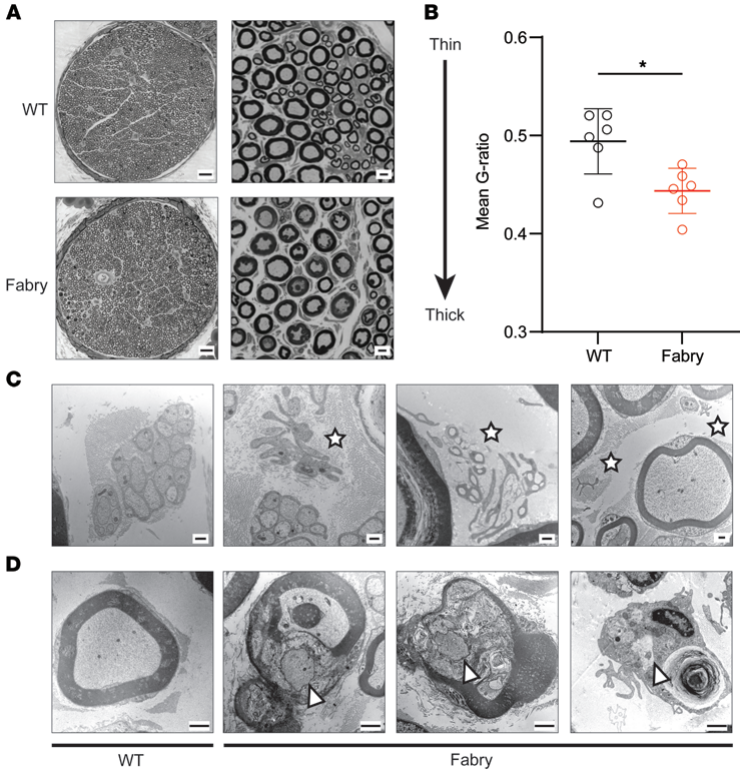
Schwann cells in Fabry sensory nerves are disrupted morphologically. (**A**) Left: Representative light microscopy images of tibial nerve from Fabry and WT rats; scale bar 50 μm. Right: Magnified images of peripheral nerve used to assess myelin architecture; scale bar 5 μm. (**B**) Analysis of mean G-ratio suggests that myelin sheath surrounding axons in Fabry nerves is thicker than myelin sheath surrounding WT axon. (**C**) Representative transmission electron microscopy images of denervated Schwann cells (stars) in the Fabry peripheral nerve, but not in the WT nerve; scale bar 0.5 μm. (**D**) Representative transmission electron microscopy images show the presence of regenerating axons within or near degenerating myelinated fibers in the Fabry saphenous nerve (arrowheads) but not in the WT nerve; scale bar 1 μm. (**A** and **B**) Tissue from *n* = 6 animals per genotype, 1 tibial nerve fascicle analyzed per animal; (**C** and **D**) tissue from *n* = 4 animals per genotype. (**B**) Reported as mean ± SEM plotted per individual animal fascicle, unpaired Student’s *t* test, * *P* < 0.05.

We asked what mechanisms may drive the hyperexcitability we observed in the sensory neuron membrane. Numerous observations indicate sensory neurons communicate with surrounding glia (see ref. 42 for a review), and clinical tissues from patients with Fabry disease show morphological changes in Schwann cells (11, 13, 18). This suggests that glial signaling may disrupt the function of sensory neurons. We first determined whether myelinating Schwann cells exhibit abnormal morphology by measuring the g-ratio (ratio of the inner axonal diameter to total outer diameter) within tibial nerve cross sections (43) (Figure 3A). G-ratios of Fabry nerves were lower than WT nerves, indicating that Fabry axons have thicker myelin sheaths than WT axons (Figure 3B and Supplemental Figure 4). To assess denervation of Schwann cells, we examined saphenous nerves from Fabry rats by electron microscopy. This revealed denervated nonmyelinating Schwann cells (Figure 3C) and evidence of regenerating axon tracks within or near degenerated myelinated axons (44) (Figure 3D). Thus, Schwann cell morphology and phenotype are disrupted in Fabry disease sensory axons.

### Fabry Schwann cell mediators sensitize rat sensory neurons.

**Figure 4:**
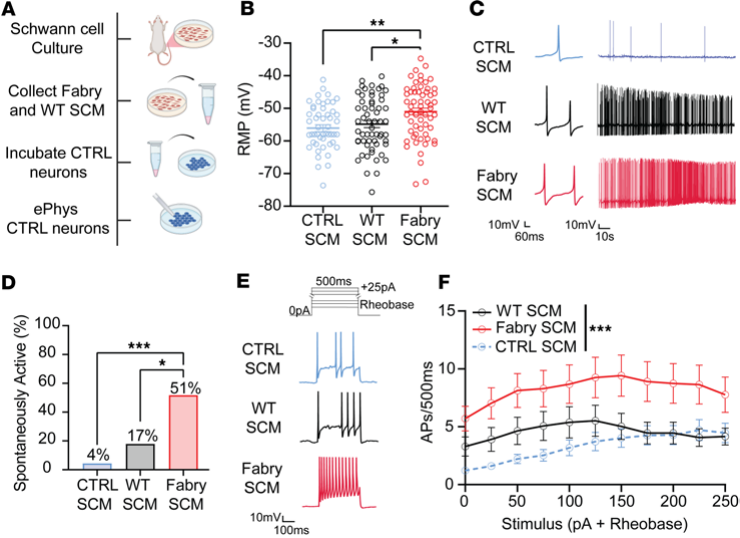
Fabry Schwann cell mediators induce peripheral neuron sensitization. (**A**) Cultured DRG neuron soma from naive Sprague Dawley (CTRL) rats were incubated with unconditioned CTRL, WT, or Fabry-SCM overnight, then washed out prior to current clamp electrophysiology recordings. (**B**) Neurons exposed to Fabry-SCM demonstrated significantly depolarized RMPs. (**C**) Representative traces of spontaneous firing from neurons incubated with CTRL, WT, and Fabry-SCM over 2 minutes. (**D**) Incubation of Fabry-SCM caused more neurons to exhibit spontaneous activity. Neurons that fired 1 or more action potentials at RMP were considered spontaneously active. (**E**) Current protocol and representative current-evoked traces from neurons exposed to CTRL-SCM, WT-SCM, or Fabry-SCM undergoing stimulation of 150 pA above rheobase for 500 ms. (**F**) Fabry-SCM enhanced the firing frequency of neurons compared with neurons exposed to WT- or CTRL-SCM. WT- and Fabry-SCM derived from *n* = 5 individual animal Schwann cell cultures per genotype. (**B**) *n* = 51–60 neurons per treatment from 21 animals; (**C** and **D**) *n* = 23–29 neurons per treatment from 8 animals; (**E** and **F**) *n* = 28–31 neurons per treatment from 13 animals. Data reported as (**B** and **F**) mean ± SEM, (**D**) mean. (**B**) One-way ANOVA with Bonferroni post hoc comparison and (**F**) 2-way repeated measures ANOVA with main effect of treatment (*** *P* < 0.0001). (**D**) χ^2^ with corrected Fisher’s exact post hoc comparison. * *P* < 0.05, ** *P* < 0.01, *** *P* < 0.001. SCM, Schwann cell–conditioned media; AP, action potential.

As Schwann cells in other neuropathic injury models release paracrine signaling compounds that alter ion channel function of sensory neurons (45–47), we further hypothesized that hyperexcitability of the Fabry sensory neuron membrane may be induced by Schwann cell–released mediators. To test this, we cultured nearly pure (>90%), viable Schwann cells isolated from sciatic nerves of Fabry or WT rats (48) (Supplemental Figure 5, A–C) and collected Schwann cell–conditioned media (SCM) 3 days after plating. Both Fabry and WT Schwann cells exhibited robust and concentration-dependent calcium influx in response to ATP, indicating the health of these cells (Supplemental Figure 5, D and E). Naive small-diameter DRG neurons were incubated with SCM from Fabry (Fabry-SCM) or WT Schwann cells, or unconditioned media (CTRL) overnight (Figure 4A). Excitability was measured using current clamp recordings 1 hour after washout. Sensory neurons treated with Fabry-SCM displayed significantly depolarized resting membrane potentials (RMPs) compared with neurons treated with either WT Schwann cell media or neurons exposed to unconditioned media (Figure 4B). Furthermore, a higher proportion of sensory neurons exposed to Fabry-SCM exhibited spontaneous firing (Figure 4, C and D), and naive neurons exposed to Fabry-SCM exhibited increased current-evoked firing frequency (Figure 4, E and F). Other passive or active membrane properties relevant to excitability, such as rheobase and action potential characteristics, were not different between treatment groups (Supplemental Table 3). These data suggest that Fabry Schwann cell–secreted factors contribute to the development of hyperexcitability observed in Fabry sensory neurons.

### Fabry Schwann cell mediators evoke peripheral hypersensitivity to light touch.

**Figure 5:**
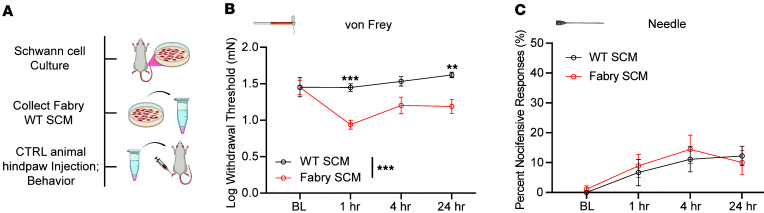
Naive rats exhibit mechanical hypersensitivity due to peripheral injection of Fabry-SCM. (**A**) Naive Sprague-Dawley rats were given intraplantar injections of WT or Fabry-SCM and underwent von Frey and needle poke stimulation of the hind paw. (**B**) Naive rats injected with Fabry-SCM exhibited increased mechanical hypersensitivity based on reduced 50% von Frey withdrawal thresholds. (**C**) Rats exposed to either WT or Fabry-SCM displayed a similar nocifensive response frequency to needle poke. (**B** and **C**) *n* = 9 animals per treatment, reported as mean ± SEM, 2-way repeated measures ANOVA main effect of treatment with Bonferroni post hoc comparison. ** *P* < 0.01, *** *P* < 0.001. BL, preinjection baseline behavioral measurements; SCM, Schwann cell–conditioned media.

Hyperexcitability and spontaneous activity of nociceptors are associated with pain behaviors (49–51). We therefore determined whether mediators released from Fabry Schwann cells sensitize peripheral nerve terminals in vivo and induce mechanical hypersensitivity, a feature often associated with evoked pain in humans (52). To test this, we injected Fabry- or WT-SCM into the glabrous skin of the hind paw of naive rats and assessed behavioral responses to mechanical stimulation (Figure 5A). Naive rats injected with Fabry-SCM displayed robust hypersensitivity to light touch by 1 hour and through 24 hours postinjection, as assessed by von Frey stimulation (Figure 5B). We also asked whether naive animals exposed to Fabry-SCM exhibit mechanical hyperalgesia to noxious needle stimulation; however, we observed no differences between treatments (Figure 5C). These data support the hypothesis that Fabry Schwann cells release mediators that sensitize peripheral nerve terminals.

### Fabry Schwann cells release p11 to sensitize sensory neurons and cause hypersensitivity to light touch.

**Figure 6:**
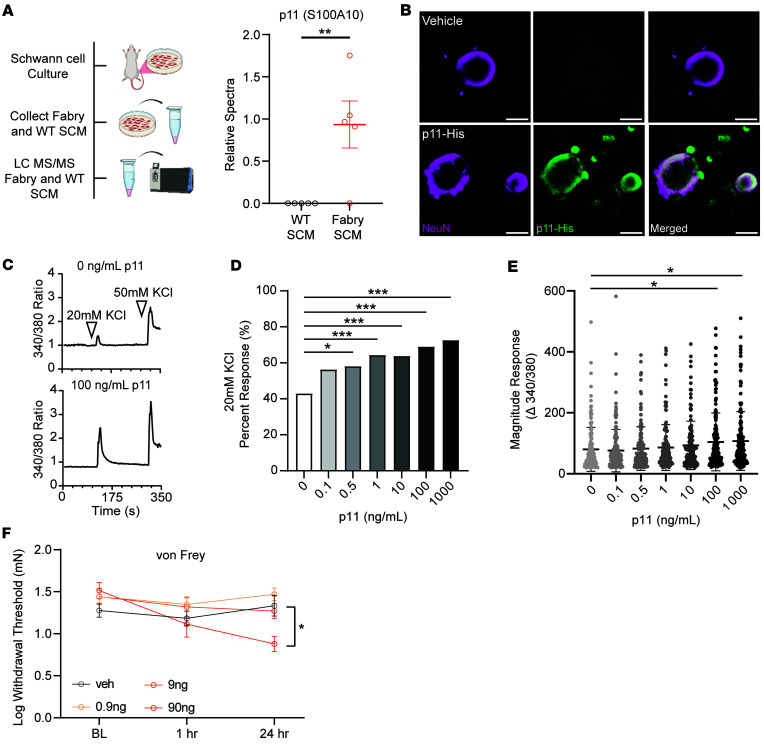
Fabry Schwann cells release the protein p11 (S100A10), which alters isolated DRG neuron function and induces pain-like behaviors in rats. (**A**) NanoLC-MS/MS analysis was performed on Fabry- and WT-SCM. Fabry-SCM contained significantly more p11 (S100-A10) compared with WT samples; refer to Supplemental Table 4 for a list of the other significantly altered proteins. (**B**) Representative immunofluorescence images of DRG neuron soma incubated with 100 ng/mL of His-tagged recombinant p11 or without p11 (CTRL) (scale bar = 20 μm). (**C**) Representative calcium imaging traces of 0 ng/mL and 100 ng/mL p11 incubated neurons exposed to 20 mM KCl for 10 seconds, 50 mM KCl used as a positive control. Traces are representative from 3 animals. (**D**) Significantly more neurons incubated with soluble p11 at various concentrations (0.5–1,000 ng/mL) responded to mild depolarization with 20 mM KCl as assessed using calcium imaging. (**E**) Neurons incubated with soluble p11 exhibited increased 20 mM KCl–induced calcium influx. (**F**) Intraplantar injection of p11 into naive Sprague-Dawley rats decreased hind paw withdrawal thresholds to von Frey stimulation. (**A**) *n* = SCM samples from 5 animals per genotype, plotted per animal; (**B**) representative images, 4 independent DRG cultures; (**C**–**E**) *n* = 140–160 neurons per treatment from 3 animals; (**F**) *n* = 8 animals per dose. (**A**, **E**, and **F**) reported as mean ± SEM, (**D**) reported as mean. (**A**) Benjamini-Hochberg–corrected 2-tailed Student’s *t* test, (**C**) χ^2^ with corrected Fisher’s exact post hoc comparison, (**E**) 1-way ANOVA with Bonferroni post hoc comparison, (**F**) 2-way repeated measures ANOVA with Bonferroni post hoc comparison. * *P* < 0.05, ** *P* < 0.01, *** *P* < 0.001. BL, preinjection baseline behavioral measurements; SCM, Schwann cell–conditioned media.

Schwann cells upregulate protein secretion during nerve injury (53–55), which we hypothesize may drive sensitization of sensory neurons. Therefore, we characterized the composition of Fabry and WT-SCM using unbiased proteomic mass spectrometry. We identified 339 proteins in Fabry and WT-SCM. Of the identified proteins, Fabry-SCM exhibited upregulation of 4 proteins and downregulation of 7 proteins (Supplemental Table 4). One of the most highly upregulated proteins in Fabry-SCM was S100A10, also known as p11 (Figure 6A). Previous literature has shown that deletion of sensory neuron p11 reduces pain-like behaviors in a spinal nerve ligation model of neuropathic pain and reduces voltage-gated sodium channel function in DRG neurons (56), suggesting that in Fabry disease, p11 may influence sensory neuron excitability (57). Sensory neurons isolated from Fabry DRGs did not contain elevated p11 compared with WT neurons (Supplemental Figure 6). We next hypothesized that DRG neurons may internalize p11 released from Fabry Schwann cells, which could underlie the hyperexcitability of both naive DRG neurons exposed to Fabry-SCM and Fabry DRG neuronal somata. To test this, we verified internalization of recombinant p11 by cultured naive DRG soma (NeuN^+^) following overnight incubation (Figure 6B). We next tested whether application of p11 alters the sensitivity of DRG neurons. We incubated cultured naive sensory neurons with various concentrations of recombinant p11 overnight. After washout, we measured calcium transients in response to exposure to a depolarizing stimulus, elevated potassium (58). Overnight incubation of 0.5–1,000 ng/mL p11 increased the proportion of DRG neurons that responded to 20 mM KCl in the bath solution (Figure 6, C and D), and incubation of 100–1,000 ng/mL p11 increased the response magnitude to 20 mM KCl (Figure 6E). We then investigated the effects of recombinant p11 on peripheral terminals in vivo by assessing mechanical withdrawal thresholds following hind paw injection of naive rats. p11 elicited a concentration-dependent hypersensitivity to light touch (Figure 6F). Thus, p11 sensitizes peripheral nerve terminals and isolates sensory neuron somata.

### p11 contributes to Schwann cell–mediated depolarization of sensory neurons in Fabry disease.

**Figure 7:**
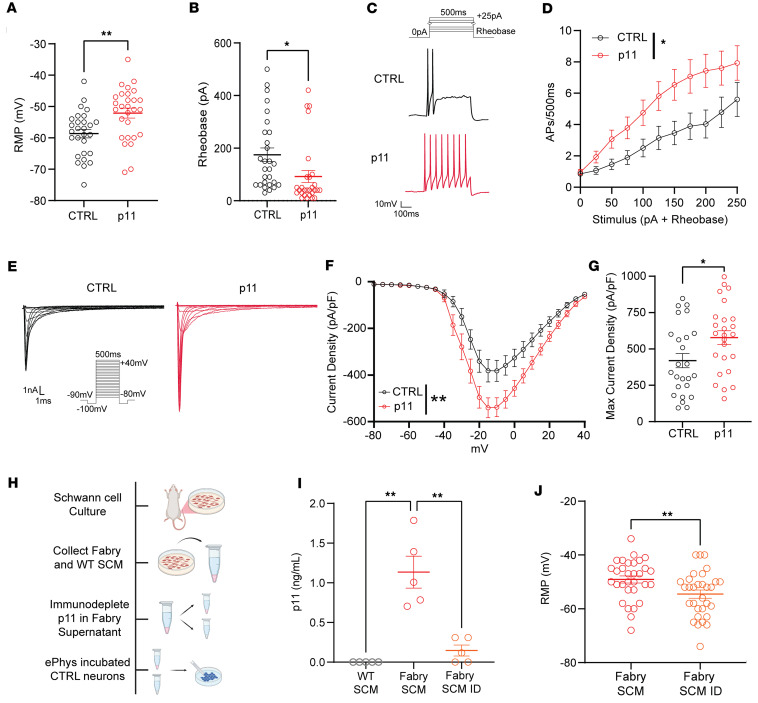
p11 contributes to the resting membrane depolarization of naive DRG neurons treated with Fabry-SCM. (**A**) Naive neurons exposed to 100 ng/mL p11 exhibited significantly depolarized RMPs. (**B**) Incubation of p11 reduced rheobase in naive neurons. (**C**) Current protocol and representative traces of neurons undergoing current stimulation of 200 pA above rheobase for 500 ms. (**D**) Neurons exposed to p11 exhibit increased firing frequency to suprathreshold current stimulation compared with CTRL. (**E** and **F**) Incubation with 100 ng/mL p11 enhanced peak sodium current densities. (**G**) The maximum inward current density was higher in neurons exposed to p11, reported as absolute values. Mean capacitance values (pF ± SEM) for neurons tested were 25.5 ± 2.0 and 23.5 ± 1.9 for CTRL and p11-incubated neurons, respectively. (**H**) p11 immunodepletion protocol from Fabry-SCM media for electrophysiology studies in naive DRG neurons. (**I**) Immunodepletion reduced p11 concentration of Fabry-SCM by 86%, with WT-SCM exhibiting concentrations below the limit of detection as measured by ELISA. (**J**) Naive neurons exposed to Fabry-SCM-ID exhibited a hyperpolarized RMP compared with neurons treated with Fabry-SCM. Values reported as mean ± SEM. (**A**–**D**) *n* = 28 neurons per treatment from 7 animals; (**E**–**G**) *n* = 26 neurons per treatment from 8 animals; (**I**) *n* = 5 WT and Fabry Schwann cell cultures from individual animals; (**J**) *n* = 31 neurons per treatment from 8 animals. (**A**, **B**, **G**, and **J**) Unpaired Student’s *t* test, (**D** and **F**) 2-way repeated measures ANOVA with main effect of treatment, (**I**) 2-way ANOVA, Bonferroni post hoc comparison. * *P* < 0.05, ** *P* < 0.01. SCM, Schwann cell–conditioned media; SCM-ID, Schwann cell–conditioned media with immunodepleted p11; AP, action potential.

As p11 sensitizes sensory neurons to depolarizing stimuli, we then asked whether p11 directly contributes to the Fabry-SCM– mediated neuronal hyperexcitability. Naive DRG neurons incubated with p11 overnight displayed significantly depolarized RMPs (Figure 7A) and decreased rheobase (Figure 7B). Furthermore, neurons fired more action potentials in response to depolarizing current injections following p11 incubation (Figure 7, C and D). Overnight incubation with p11 caused a strong but statistically insignificant increase in the proportion of neurons exhibiting spontaneous activity (Supplemental Figure 7, A–C). Other membrane properties related to excitability remained similar between treatments (Supplemental Table 5). Prior literature shows that deletion of neuronal p11 reduces voltage-gated sodium channel function in DRG neuron somata (56), which may influence excitability (57). Thus, we assessed voltage-gated sodium channel function by performing voltage clamp recordings of naive sensory neurons incubated overnight with p11 (Figure 7, E–G). Neurons exposed to p11 exhibited increased voltage-dependent sodium channel current densities compared with control neurons (Figure 7, F and G).

We then determined whether p11 is a critical compound released from Fabry Schwann cells to induce sensory neuron hyperexcitability. To test this, we collected Fabry SCM and immunodepleted p11 using Dynabead-conjugated anti-p11 antibody and magnetic sorting (Figure 7H). Depletion of p11 was confirmed by ELISA (Figure 7I). The concentration of p11 in the immunodepleted SCM (mean 0.15 ng/mL) was below the effective concentration in which p11 induced significant alteration to neuronal activity (Figure 6, C–E). Therefore, we incubated naive sensory neurons with either complete Fabry-SCM or Fabry-SCM with immunodepleted p11 (Fabry-SCM-ID); we then used current clamp electrophysiology to detect differences in spontaneous activity or excitability. Neurons incubated with Fabry-SCM-ID exhibited a significantly hyperpolarized RMP relative to standard Fabry-SCM (Figure 7J). However, removal of p11 did not affect the proportion of neurons that exhibited spontaneous firing, rheobase, or current-evoked firing frequency (Supplemental Figure 8, A–C). Additionally, other passive and current-evoked membrane properties related to excitability were similar between Fabry-SCM and Fabry-SCM-ID cohorts (Supplemental Table 6). These findings suggest that release of p11 from Fabry Schwann cells facilitates resting membrane depolarization of DRG neurons and may contribute to the hyperexcitability of sensory neurons in Fabry disease rats.

## Discussion

Patients with Fabry disease experience chronic and episodic pain. The mechanisms underlying Fabry pain have received little research attention. Here, we used a genetic rat model of Fabry disease to show that peripheral sensory neurons display prominent, persistent spontaneous activity and hyperexcitability. Moreover, Schwann cell morphology is known to be disrupted in peripheral nerves of patients with Fabry disease and Fabry rats. Here, we demonstrate that these disrupted Schwann cells contribute to the pathological hyperexcitability in Fabry sensory neurons by releasing factors that include p11 (S100A10). Together, our findings suggest that pathological communication between Schwann cells and sensory neurons in the peripheral nerve may contribute to the chronic pain in Fabry disease.

Patients with Fabry disease experience many pain phenotypes that begin in childhood, including debilitating shooting and burning pain (4, 59, 60). Previously, we showed that Fabry rats display mechanically evoked (22) and spontaneous pain (23) phenotypes that are similar to patients. These pain phenotypes have been shown to be caused by increased nociceptor excitability in other neuropathic pain disorders (61, 62), but nociceptor excitability has not been examined in Fabry rats. Here, we show that sensory neurons in Fabry rats exhibit elevated spontaneous activity and increased mechanical sensitivity. Furthermore, C-, Aδ-, and Aβ-fibers in Fabry rats displayed elevated firing frequency in response to punctate mechanical stimuli. These data are consistent with our previous finding that showed small-diameter sensory neurons are sensitized to mechanical stimuli applied to the soma membrane (22). Here, we also show that small-diameter sensory neurons exhibit current-evoked hyperexcitability, indicating that increased intrinsic excitability may underlie both the spontaneous activity and the mechanical sensitization of peripheral sensory neurons in Fabry rats. Together, these data suggest that peripheral sensory neurons are a critical site of pathology that drives the chronic pain in Fabry rats and support the use of the Fabry rat to study mechanisms that underlie chronic pain in Fabry disease.

Our findings in Fabry rats diverge from those in a mouse model of Fabry disease (Gla^−/0^) (63). Fabry mice exhibited no change in C-fiber sensitivity to mechanical stimulation of the hind paw, and Aδ-fibers displayed decreased sensitivity to mechanical stimulation in ex vivo saphenous nerve recordings (64). The same mouse model also showed decreased DRG neuron excitability and decreased voltage-gated sodium and calcium channel conductance (64, 65). Because patients show evidence of peripheral neuron hyperexcitability (64, 66), we suggest that the Fabry rat may better recapitulate sensory neuron dysfunction in patients than existing Fabry mouse models. On the other hand, the Fabry rat exhibits some phenotypes that may not reflect some symptoms of chronic pain in patients. For example, our previous work showed that Fabry rats do not have altered behavioral heat sensitivity (22); in contrast, Fabry mouse models (67) and patients with Fabry disease (13) have hyposensitivity to heat. Consistent with this, Fabry patient–derived induced sensory neurons also show hypoexcitability to heated buffer (68). A second example is that Aβ-fibers from Fabry rats exhibit significant hypersensitivity to mechanical stimulation (Figure 1, I and M); yet, it has been predicted that Aβ-fibers in patients would be less sensitive to mechanical stimuli as patients display increased thresholds (reflecting decreased sensitivity) to vibratory stimuli (6, 13). In our study, Aβ-fibers in Fabry rats were assessed by punctate — not vibratory — mechanical stimuli; therefore, the effects of Fabry disease on rapidly adapting and slowly adapting myelinated fibers may differ. The nuances in different studies performed on various rodent models of Fabry disease should be carefully considered when comparing these preclinical studies to patient phenotypes.

Peripheral nerves from patients with Fabry disease show evidence of denervated nonmyelinating Schwann cells and Schwann cells without axon association (69). Peripheral nerves from Fabry rats exhibit pathologies related to the Remak bundle, including larger axon diameters (36) and decreased unmyelinated fiber densities in peripheral nerves (36). Here, we show evidence of demyelinated Schwann cells in the saphenous nerve of Fabry rats (Figure 3, C and D). Denervated Schwann cells migrate, form regenerative tracks to facilitate directional axon growth through release of growth factors, and are a key characteristic of nerves undergoing Wallerian degeneration (44, 70–72).

Exciting emerging evidence indicates that Schwann cell dysfunction contributes to the development and maintenance of chronic pain. Optogenetic or chemogenetic activation of Schwann cells induces peripheral sensory nerve sensitization and pain-like behaviors in rodents (19–21), and Schwann cells have been shown to release specific algogens that elicit pain behaviors in rodents (45–47). Here, we have identified a mechanism in Fabry disease by which Schwann cells induce nociceptive neuron hyperexcitability by releasing secreted factors. Sensory neuron somata isolated from naive rats exposed to Fabry-SCM developed hyperexcitability, including increased spontaneous activity and increased current-evoked firing frequency. Furthermore, injection of Fabry-SCM into the hind paw of naive rats induced mechanical hypersensitivity, suggesting that the mediators released from Fabry Schwann cells can activate the peripheral terminals of cutaneous sensory afferent neurons. It is known that Schwann cells can induce gene expression changes in adjacent sensory neurons (73) to alter the expression and function of ion channels such as voltage-gated sodium channels that are relevant for neuron excitability (74–77). Our findings here suggest that in Fabry disease, Schwann cells contribute to the enhanced excitability of peripheral sensory neurons by secreting mediators that alter neuronal genes and ion channel function.

Here, we used an unbiased screening method to identify putative algogenic mediators released from Schwann cells. We identified that Fabry Schwann cells increase their release of the protein p11 (S100A10). We also demonstrated that p11 induces likely previously uncharacterized regulation of nociceptive signaling, including enhanced sensory neuron excitability and induction of mechanical hypersensitivity, when peripherally injected into naive rats. Little is known about the paracrine signaling mechanisms by which p11 induces sensory neuron hyperexcitability. P11 is expressed by many cell types and is associated with exosomal secretion (78); thus, exosomal secretion is a potential mechanism for release of p11 from Fabry Schwann cells. In neurons, intracellular p11 has also been shown to modulate the trafficking and plasma membrane localization of many ion channels that are relevant for excitability (reviewed in ref. 79), including the voltage-gated sodium channel Nav1.8 (56, 80, 81). Our findings show that exogenous p11 enhances voltage-gated sodium channel currents (Figure 7, E–G); this finding is consistent with a previous study that demonstrated voltage-gated sodium channel currents are reduced in neurons following p11 knockdown (56). Furthermore, we found that incubation of naive sensory neurons with p11 enhances calcium influx in response to KCl (Figure 6, C–E), a result that may be due to p11-induced increased membrane localization of L-type calcium channels (82, 83). Selective removal of p11 from the Fabry-SCM induced hyperpolarization of the RMP when compared with neurons treated with complete Fabry-SCM (Figure 7J, and Supplemental Table 6). This finding suggests that elevated p11 depolarizes the RMP of neurons, possibly by increasing hyperpolarization-activated cyclic nucleotide-gated (HCN) or decreasing 2-pore domain (K2P) TASK1 membrane localization (84); indeed, previous studies have shown that knockdown of p11 in neurons reduces HCN channel-mediated currents (85) and increases putative TASK1 currents (86). Together, these data suggest that Fabry Schwann cells secrete p11 that, in turn, alters the localization of various ion channels and, thereby, increases the excitability of sensory neurons.

There are limitations to this study. We have shown that mediators from Fabry Schwann cells induce mechanical hypersensitivity in naive rats and induce dysfunction of naive sensory neurons; we have not determined whether Schwann cells drive these observations in vivo in Fabry rats. In vivo experiments will be difficult since systemic knockdown of p11 would likely induce off-target effects (81), and functional neutralization of p11 released from Schwann cells in vivo is technically challenging. Future studies can be performed in vivo. Furthermore, it is difficult to determine the relative effects of Schwann cell–released p11 compared with intrinsic neuronal p11 expression on altered DRG neuron physiology in vivo. Fabry DRG neurons have similar expression of p11 compared to WT (Supplemental Figure 6). It is unclear whether the localization, function, or uptake of neuronal p11 in Fabry DRG neurons is altered, in addition to the increased p11 release by Fabry Schwann cells. Another limitation is that Fabry DRG neurons exhibit membrane properties related to hyperexcitability (Figure 2) that are different from naive neurons incubated with Fabry-SCM (Figure 4) or with p11 (Figure 7, A–D). It is possible that in vitro application of Fabry Schwann cell mediators on DRG neurons does not ideally recapitulate the sensitization observed in Fabry DRG neurons in vivo. Alternatively, chronic glycosphingolipid accumulation in Fabry sensory neurons (22, 36) and glycosphingolipid-independent consequences of α-Gal A deficiency (87) may alter neuron function. An additional limitation is that removal of p11 from Fabry-SCM caused a hyperpolarization of the RMP (Figure 7, H–J), suggesting that other factors released from Fabry Schwann cells also alter the excitability of naive neurons incubated with complete Fabry-SCM (Figure 4). It is curious that removal of p11 from the Fabry-SCM only hyperpolarized the neuronal RMP without inducing associated changes to membrane properties that underlie excitability, such as rheobase. This may be due to potential collateral depletion of p11-bound proteins (Figure 7, H–J). Notably, p11 binds other proteins to prevent intracellular degradation (88), and these proteins may also influence ion channel function. To ideally address these discrepancies, future approaches could inhibit release of specific mediators from Fabry Schwann cells in vivo.

In summary, this study shows that peripheral nerve dysfunction and sensory neuronal hyperexcitability underlie the chronic pain behavior in this rat model of Fabry disease and that sensory neuron hyperexcitability in Fabry disease may result from chronically altered Schwann cell signaling, potentially involving released p11 that signals to sensory neurons. Further studies are warranted to investigate both the peripheral sensory neuron hypersensitivity and glial cell dysfunction in Fabry disease to potentially discover novel therapeutic targets for this debilitating and understudied genetic pain disorder.

## Methods

### Sex as a biological variable

As Fabry disease is an X-linked genetic disorder predominantly affecting males and our Fabry disease rat model displays more robust sensory phenotypes in males (22), all cellular, histological, and electrophysiological studies with Fabry rats in this study exclusively used males. Experiments using SCM in Sprague-Dawley rats used both male and female rats to account for sex as a biological variable.

### Animal model

The X-linked genetic Fabry disease rat model (22) (Rat Genome Database symbol: Glaem2Mcwi) was used for in vivo dorsal root teased single-unit recordings, provided primary sensory neuron and Schwann cell cultures, and was compared with age- and sex-matched WT littermate controls. All rats used for in vivo and in vitro electrophysiology and collection of Fabry Schwann cells were hemizygous males between 20 and 40 weeks old. For evaluating the effects of Schwann cell media or p11 protein treatment on naive sensory neuron function, male Sprague-Dawley outbred rats (Envigo) aged 15–30 weeks were used (CTRL). For animal behavior experiments, both male and female Sprague-Dawley rats from Taconic were used. As no sex differences were observed in behavior studies, sexes were combined for analysis.

### Light microscopy and transmission electron microscopy

#### Peripheral nerves.

Nerves from Fabry and WT rats were collected (36) and processed (89, 90) as previously described. The relevant method details are presented in the Supplemental Methods.

#### DRGs.

Tissue from Fabry and WT rats aged 13 weeks was harvested and processed as previously described (22, 91). The relevant method details are presented in the Supplemental Methods.

### Cell culture

#### Sensory neuron soma.

DRG neurons were harvested, dissociated, and cultured as previously described (22, 92). Further details are presented in the Supplemental Methods.

#### Schwann cells.

Schwann cells were cultured using a previously published protocol (48) with modifications. Further details are presented in the Supplemental Methods.

#### Schwann cell culture purity.

Schwann cell culture purity was assessed using immunofluorescence of the Schwann cell marker SOX10 (93) and DAPI to mark all cells. Analysis was done using a modified colocalization analysis as we previously described (36). Further details are presented in the Supplemental Methods.

#### SCM.

Schwann cell cultures were grown for 2 days in Schwann cell growth media to 70%–80% confluence. Cells were then washed with PBS and replaced with Schwann cell collection media (high-glucose DMEM [Thermo Fisher Scientific], 10 nM neuregulin [Recombinant Heregulin-β1177–244, PeproTech], and 2 μM forskolin [MilliporeSigma]) and cultured for 1–2 days. Schwann cells have been shown to grow in culture with or without crude serum (94); serum was not included in collection media to reduce confounding variables for all experiments. These media were collected, filtered using a 0.22 μm filter (CELLTREAT) to remove debris or cell fragments, flash-frozen in liquid nitrogen, and stored at –20°C or –80°C for less than 1 month for functional assays. Media samples, represented as either SCM or unconditioned media controls, were then thawed and used for all subsequent experiments. Media underwent a maximum of 2 flash-freeze cycles for all assessments and were not diluted.

#### p11 treatment.

Recombinant rat protein p11 with an N-terminal His Tag (S100A10 Recombinant Protein, Aviva Systems Biology, OPCD06771) was dissolved in distilled water to obtain a final concentration of 100 μg/mL and stored at –80°C for less than 1 month. Protein was further diluted in DRG neuron culture media to obtain the respective concentrations (0.1–1,000 ng/mL) for calcium imaging experiments and whole-cell patch clamp electrophysiology experiments; neurons were incubated with this protein overnight. For intraplantar injection to assess rodent behavior, protein was dissolved in saline to a dose of 0.9, 9, or 90 ng per 30 μL injection; saline was used as vehicle injections.

#### p11 immunofluorescence.

Sensory neuron soma isolated from naive rats were cultured and treated with or without 100 ng/mL recombinant rat protein p11 overnight followed by washout. Cells were fixed and underwent an immunofluorescence protocol as detailed in the Supplemental Methods. A negative control stain was also analyzed (p11 no Ig), which consisted of cultures that underwent exposure to 100 ng/mL p11 and the subsequent immunofluorescence protocol without inclusion of the anti-His tag primary antibody.

#### p11 ELISA of isolated DRG neurons.

Details are presented in the Supplemental Methods.

#### p11 immunodepletion.

Fabry-SCM were collected as described above. Media were split into 2 equal aliquots: one undergoing immunodepletion and the other used as a control batch. Anti-p11 antibody (10 μg) (S100A10 polyclonal antibody, ProteinTech, 11250-1-AP) was incubated with 50 μL Dynabeads Protein A (Invitrogen) for 10 minutes, and supernatant was removed with a DynaMag-2 Magnet tube rack (Invitrogen). Afterward, Fabry-SCM was added the anti-p11 antibody–bound Dynabeads and left to incubate on a rotating platform for 60 minutes at room temperature. Samples were then placed onto the magnetic tube rack and supernatant was removed. Depletion of p11 from the media was verified using Rat S100 Calcium Binding Protein A10 (S100A10) ELISA Kit (Biomatik, EKN48271-96T) as per the included instructions.

### Dorsal root teased-fiber single-unit recording

Fabry and WT rats underwent in vivo dorsal root single-unit recordings as we reported previously (97). Briefly, rats were anesthetized with subcutaneous injection of urethane (100 mg/kg) followed by isoflurane that was progressively reduced to 0.2% over 30 minutes. A laminectomy was performed to expose the spinal cord from the T13 to the L3 vertebrae, which was covered with warm mineral oil (36°C). Dura was removed, and rats were mounted on a spinal frame with stabilizing vertebral clamps. The L4 dorsal root was gently released from connective tissues and transected at the rootlets adjacent to the spinal cord. The dorsal root was placed onto a glass platform and teased into fine bundles that were individually placed onto a platinum/iridium recording electrode for observation of spontaneous and evoked activity. A reference electrode was placed in adjacent muscle tissue. Signals were collected with an Axoclamp 900 A microelectrode amplifier (Molecular Devices) with the headstage (HS-9A-x0.1U with feedback resistance of 100 MΩ) serving as a preamplifier with gain setting of 500 or higher, were filtered at 1 kHz, and were sampled at 10 kHz using a digitizer (DigiData 1440 A, Molecular Devices). Action potentials were isolated by setting the threshold above background noise.

For recording spontaneous activity, bundles were observed for a 3-minute observation period and recorded for 3–4 minutes if spontaneous activity was present. For recording evoked activity, the receptor field of a unit was identified by low-intensity mechanical stimulation of the glabrous plantar skin of the hind paw with a small glass probe (with 1 mm round tip). Firing frequency to innocuous mechanical stimulation was then examined with graded von Frey monofilaments and a modified von Frey monofilament with a tungsten tip for noxious stimulation for 10 seconds. Prior to evoked stimulation, fibers were assessed for spontaneous activity for 10 seconds, and the mean firing frequency of this spontaneous activity was subtracted from the observed firing frequency during evoked stimulation. To identify unit types, the sciatic nerve was stimulated and used to calculate conduction velocity by dividing the distance between stimulation and recording sites by the response latency of the electrically evoked action potential. Fiber types were then classified based on conduction velocity; 20 m/s or greater for Aβ, between 3 and 20 m/s for Aδ, and less than 3 m/s for C-type. The genotype was blinded for all analyses.

### Whole-cell patch clamp electrophysiology

Neuronal soma were categorized into either small (≤32 μm) or large diameter (>32 μm), as the electrophysiology properties of rodent neurons vary based upon size (95, 96), and the majority of small-diameter neurons are considered nociceptors (97). Neuronal capacitance was fully compensated and continuously monitored to ensure stable recording conditions. Whole-cell recordings were obtained using HEKA EPC10 amplifier, and recordings were obtained using Patchmaster Next software (version 1.2). The genotype and treatment were blinded for all analyses. All reagents for patch clamp analysis were obtained from Thermo Fisher Scientific unless otherwise specified.

#### Current clamp recordings.

Isolated sensory neuron soma were superfused with extracellular buffer (140 NaCl, 2.8 KCl, 2 CaCl_2_, 1 MgCl_2_, 10 HEPES, 10 glucose, and 8.8 sucrose, pH 7.4 ± 0.02 and 310 ± 3 mOsm, adjusted with sucrose). Borosilicate glass pipettes (2–6 MΩ) filled with internal solution (in mM: 135 KCl, 4.1 MgCl_2_, 2 EGTA, 0.2 GTP, 2.5 ATP, and 10 HEPES, pH 7.2 ± 0.02 and 290 ± 2 mOsm) were pulled using a Sutter Instruments P87 pipette puller and used to perform patch clamp recordings. Series resistance was maintained at less than 15 MΩ and compensated at 80%.

#### Spontaneous activity.

Whole-cell recordings were established in voltage clamp mode, then switched to current clamp mode to measure RMP. Voltage was recorded at RMP for 2 minutes to observe spontaneous, suprathreshold action potentials (>0 mV) with 0 current injection. Cells firing at least 1 action potential during the 2-minute period were considered spontaneously active.

#### Current-evoked excitability.

Neuron soma were held at –70 mV to prevent spontaneous activity from influencing current-evoked recordings, and intrinsic excitability was recorded using the following protocols (98): (i) Voltage-current (V-I) relations were obtained using 20 sweeps of 500 ms alternating ascending/descending current pulses (5 pA steps from holding current). The plateau voltage deflection was plotted against current amplitude, and input resistance was determined from the slope of a V-I plot. (ii) Action potential (AP) properties were measured using an ascending series of 500 ms depolarizing current pulses. Rheobase was defined as the minimal current to elicit at least a single spike. AP threshold was determined from a derivative function, where *dV/dt* first exceeded 28 mV/ms. AP amplitude was determined relative to AP threshold, and AP half-width was measured as the width at half of the AP amplitude. (iii) A series of eleven 500 ms depolarizing current steps (range, rheobase to 250 pA above rheobase; 25 pA increments, 20-second intervals) or seven 500 ms depolarizing current steps (range, rheobase to 1,600 pA above rheobase; 200 pA increments, 20-second intervals) was used to determine AP firing frequency.

#### Voltage clamp recordings.

Extracellular buffer (in mM: 70 NaCl, 70 choline-Cl, 3 KCl, 1 MgCl_2_, 1 CaCl_2_, 10 glucose, 10 HEPES, pH 7.35 ± 0.02 and 310 ± 3 mOsm, adjusted with sucrose) was flowed over isolated sensory neuron soma from control rats. The addition of 20 mM TEA-Cl and 0.1 mM CdCl_2_ was added to extracellular buffer to block voltage-gated K^+^ channels and Ca^2+^ channels, respectively (99). Fire-polished borosilicate glass pipettes (1–4 MΩ) were filled with internal solution (in mM: 140 mM CsF, 10 mM NaCl, 2 mM MgCl_2_, 0.1 CaCl_2_, 1.1 EGDA, 10 HEPES, pH 7.3 ± 0.02 and 310 ± 3 mOsm, adjusted with sucrose) and pulled using a Sutter Instruments P87 pipette. Soma were established in voltage clamp at a holding potential of –90 mV. Series resistance was maintained at less than 10 MΩ and compensated on 85%, then held at holding potential for 2–4 minutes. Currents were elicited by incremental depolarizing steps (+5 mV increments, 500 ms duration, 5-second intervals) between –80 mV and +40 mV, with a –100 mV hyperpolarizing pulse given before and after each step for 50 ms. The average of 3 sweeps per neuron was taken for determination of peak current density at each step, which were filtered at 2.9 kHz.

### Calcium imaging

#### Sensory neuron soma.

Calcium imaging of dissociated neuronal soma was conducted as we have previously published (92). Soma dissociated from DRGs were incubated overnight in media with or without 0.1-1,000 ng/mL p11. Soma were then washed with extracellular buffer solution (150 mM NaCl, 10 mM HEPES, 8 mM glucose, 5.6 mM KCl, 2 mM CaCl_2_, 1 mM MgCl_2_, pH 7.40 ± 0.03, and 320 ± 3 mOsm) for 30 minutes, incubated with 2.5 mg/mL Fura-2-AM (Life Technologies) for 45 minutes, and washed for 30 minutes. Fluorescence images were captured at 340 nm and 380 nm with a cooled Andor Zyla-SCMOS camera (Oxford Instruments) to calculate the bound to unbound ratio (340/380). NIS-Elements software (Nikon) was used to detect and analyze intracellular calcium changes. To induce membrane depolarization, we added 20 mM KCl to the extracellular buffer solution, while reducing NaCl concentration to maintain an osmolarity of 320 mOsm. The depolarizing solution was applied to neurons for 10 seconds to determine both number of responding neurons and response magnitude. Soma that exhibited a ≥20% increase in 340/380 ratio within 30 seconds after KCl application compared with baseline ratio were considered positive responders. As a positive control, 50 mM KCl was applied near the end of the recordings; soma were considered viable and subsequently analyzed only if they responded to 50 mM KCl. The genotype and treatment were blinded during all calcium imaging analyses.

#### Schwann cells.

A detailed description of the calcium imaging of Schwann cells is presented in the Supplemental Methods.

### Mass spectrometry

Peptides were analyzed by nanoLC-MS/MS, and detailed methods are presented in the Supplemental Methods.

### Animal behavior

Rat plantar cutaneous mechanical sensitivity was assessed as previously reported (22). Rats underwent 30 μL intraplantar (footpad) injections of saline or treatment with undiluted Schwann cell collection media or p11 (0.9, 9, and 90 ng) and were acclimated on top of a wire mesh for 1 hour, with the experimenter present for 30 minutes of this period. Testing was performed at approximately the same time each day. Mechanical sensitivity threshold was determined with von Frey filaments (up-down method; ref. 100), and values were analyzed after log transforming (101). Hypersensitivity to noxious force (hyperalgesia), which is selectively associated with aversion (102), was tested by needle prick (103). The treatment was blinded for all experiments. For baseline von Frey withdrawal threshold measurements, animals underwent testing within 7 days of treatment administration.

### Statistics

Results were considered statistically significant when *P* < 0.05. All data were analyzed using GraphPad Prism (Version 9.0.0). No potential outliers were removed during data analysis for this study. Statistical analyses used for each data set are indicated within each figure legend. For dorsal root teased-fiber single-unit recording, data were analyzed using χ^2^ for spontaneous activity per fiber bundle and an unpaired 2-tailed Student’s *t* test for spontaneous activity per animal and firing rate. Mechanical sensitivity to graded von Frey stimulation was analyzed using a 2-way repeated measures ANOVA, and sensitivity to needle was analyzed using an unpaired 2-tailed Student’s *t* test. For analysis of myelin pathology, data were analyzed using unpaired 2-tailed Student’s *t* test. For current clamp experiments, membrane and AP properties were analyzed using a 1-way ANOVA for testing Schwann cell media effects on neuron function and unpaired 2-tailed Student’s *t* test for testing the effects of p11 on neuron function; current-evoked firing frequency was analyzed using a 2-way repeated measures ANOVA. Percentage of spontaneously active cells was analyzed using χ^2^. For calcium imaging experiments, proportion of cells responding was analyzed using χ^2^, while response magnitude data were analyzed using a 1-way ANOVA. For voltage clamp experiments, current densities were measured using a 2-way repeated measures ANOVA, and maximum current density was analyzed using unpaired 2-tailed Student’s *t* test. For nanoLC-MS/MS, data were analyzed using a Benjamini-Hochberg corrected 2-tailed *t* test. For ELISA experiments, data were analyzed using a 2-way ANOVA. For von Frey and noxious needle tests, data were analyzed using a 2-way repeated measures ANOVA. Bonferroni post hoc corrections were used for significant ANOVAs. Fisher’s exact tests were used for significant χ^2^ tests.

### Study approval

All protocols were in accordance with NIH guidelines and were approved by the Institutional Animal Care and Use Committee at the Medical College of Wisconsin.

### Data availability

All data are available from the Supporting Data Values XLS file and from the corresponding author.

## Author contributions

TBW and CLS conceptualized the experiments. TBW, DC, VLE, EI, and BP developed the methodology. TBW, DC, EKP, and JDE performed the experiments. TBW and DC analyzed the data. TBW created the original draft manuscript. TBW, JDE, and BSD created figure visualizations. All authors contributed to the editing of the paper. Supervision was provided by CLS, BP, QHH, and NMD. Funding for this work was acquired by CLS, NMD, and TBW.

## Supplementary Material

Supplemental data

Supporting data values

## Version 1 04/22/2024

Electronic publication
