# Supplementary material for: Fabry disease Schwann cells release p11 to induce sensory neuron hyperactivity
Source: JCI Insight. 2024 Apr 22;9(8):e172869. doi: 10.1172/jci.insight.172869 (PMC11141882; doi:10.1172/jci.insight.172869)
Supplement: Supplemental data [file jciinsight-9-172869-s129.pdf]

Supplemental Methods:

<u>Light microscopy (LM) and transmission electron microscopy (TEM):</u>

<u>Peripheral nerves:</u> Nerves from Fabry and WT rats were collected (1) and processed (2, 3) as previously described. Briefly, nerves were fixed with 4% paraformaldehyde and 2% glutaraldehyde in sodium cocodylate buffer at 4°C overnight, then postfixed with 1% osmium tetroxide for 2 hours at room temperature. Samples were dehydrated in a series of methanol gradients and infused with epon. Sections 0.5 μm thick were cut for light microscopy analysis and stained with toluidine blue, while thin sections of 70 nm were prepared for transmission electron microscopy (TEM), stained with 25% uranyl acetate and counterstained with lead citrate. Cross-sections of the tibial nerve were examined using light microscopy to manually assess myelination through G-ratio analysis (quantification of inner axonal diameter to total outer diameter). Axons within the entire nerve fascicle were counted per animal and analyzed using ImageJ software. TEM cross-sections of the saphenous nerve were obtained using a JEOL 1400 Flash electron microscope operating at 80KV and assessed for morphological abnormalities. G-ratio calculation and TEM image acquisition were performed by researchers blinded to genotype.

<u>Dorsal root ganglia (DRG):</u> DRG were fixed with 10% neutral-buffered formalin and then paraffin-embedded. Blocks were cross-sectioned 4 μm thick, deparaffinized, and then stained with hematoxylin and eosin (H&E). Slides were imaged using a Hamamatsu (Hamamatsu, Japan) NanoZoomer 2.0-HT slide scanner. Neuron somata from the entire DRG cross-section were counted compared to the cross-sectional area of the DRG, and DRG were assessed for the presence of Nageotte nodules, or residual clusters of satellite glial cells associated with sensory neuron somata degeneration (4). All analyzes were performed by researchers blinded to genotype.

<u>Cell culture:</u>

<u>Sensory neuron soma:</u> Rats were anesthetized (4% isoflurane), sacrificed, and lumbar DRGs were isolated. DRGs were incubated with 1mg/ml collagenase type IV (Sigma) for 45 min then 0.05% trypsin (Sigma) for 45 min. Neuronal soma were mechanically dissociated and plated onto laminin-coated coverslips. One hour after plating, neurons were fed cell media [high glucose DMEM, 2 mmol/L L-glutamine, 1% glucose, 100 units/mL penicillin, 100 ug/mL streptomycin, 2% heat-inactivated horse serum]. Neurons grew overnight at 37°C and 5% CO2. For experiments mentioned in the text, neurons were fed with Schwann cell collection media [high glucose

DMEM (ThermoFisher), 10 nM neuregulin (Recombinant Heregulin-β1177–244, PreproTech), and 2 μM forskolin (Sigma)] that was extracted from Fabry or WT Schwann cell cultures. For some experiments, the inclusion of an unconditioned media control (CTRL) was incubated onto neurons overnight (12-16 hours), which was unconditioned Schwann cell collection media.

Schwann cells: Schwann cells were cultured using a previously published protocol (1) with modifications. Briefly, adult Fabry or WT rats were anesthetized, culled, and sciatic nerves were removed by transection under the gastrocnemius muscle and at the nerve trifurcation. The epineurium was removed, and nerve fascicles teased. These were subjected to enzymatic dissociation with 0.25% Dispase I (ThermoFisher) and 0.05% type I collagenase (EMD Milipore)) in high-glucose DMEM (ThermoFisher) with antibiotics overnight at 37 °C and 5% CO2. Enzymatic dissociation was stopped with the addition of 40% fetal bovine serum, centrifuged, and cell pellets were resuspended in DMEM with 10% serum. Resuspended cells were mechanically dissociated, then plated onto air-dried PDL (ThermoFisher) and laminin-coated plates (Sigma). Two to three hours after plating, Schwann cells were washed with PBS to remove excess debris, and then fed with Schwann cell growth media [high glucose DMEM (ThermoFisher),10 nM neuregulin (Recombinant Heregulin-β1177–244, PreproTech), and 2 μM forskolin (Sigma), 10% FBS (ThermoFisher)]. To assess Schwann cell viability, cells were un-adhered with 0.05% trypsin then diluted 1:1 in trypan blue (Sigma) and viability was automatically counted (Countess 3, Invitrogen).

Schwann cell culture purity: Schwann cells were cultured for 2 days and then fixed in 4% PFA for 20 minutes, washed with PBS, then permeabilized with blocking solution (0.05 M Tris-buffered saline with 0.3% Triton-X) for 1 hr then washed. Recombinant anti-SOX10 antibody (Abcam, EPR4007) in blocking solution was added for 1 hr (1:500), then washed with PBS and secondary antibody in blocking solution (Alexa Fluor™ 594 donkey anti-rabbit IgG, ThermoFisher, R37119) was added (1:1000) for 1 h. After washing, coverslips were mounted with mounting media + DAPI (Vector Laboratories) and imaged using a Leica SP8 Upright Confocal Microscope. The middle of the coverslip was found, and one 4x4 stitched image (16 separate images) was taken from each culture at 20x magnification. Purity of the culture was quantified using a modified colocalization analysis we have previously described [1]. Briefly, 30 DAPI+ cells that were determined to be negative for SOX10 were manually assessed for mean SOX10 fluorescence or baseline mean signal. Images of each culture were automatically assessed with an ImageJ macro. Cells were considered SOX10 positive if the fluorescence for the SOX10 signal

was four standard deviations above the baseline mean. Ratio of SOX10+ and DAPI+ cells compared to all DAPI+ cells was automatically calculated and reported as percent purity. Researchers were blinded to genotype for analyses.

p11 immunofluorescence: Sensory neuron somata isolated from naïve rats were cultured using the protocol mentioned in the Methods section and treated with or without recombinant rat protein p11 overnight with washout. Cultures were then fixed in 4% PFA for 20 minutes, washed with PBS, then permeabilized with blocking solution (0.05 M Tris-buffered saline with 0.3% Triton-X) for 1 hour then washed. 6x-His Tag Polyclonal Antibody (Invitrogen, PA1-983B, 1:200) and Anti-NeuN polyclonal (Sigma-Aldrich, ABN90, 1:800) in blocking solution was added and incubated overnight (12 hr). After wash, the secondary antibody for the 6x-His tag antibody (Donkey anti-Rabbit IgG (H+L), Alexa Fluor™ 488, ThermoFisher, A-21206, 1:1000) and for the Anti-NeuN antibody (Goat Anti-Guinea Pig IgG (H+L), Alexa Fluor® 647, Jackson, AB_2337446, 1:1000) in blocking solution were added for 1 hour. After wash, samples were mounted with ProLong™ Gold Antifade Mountant (Thermo Fisher Scientific, P10144), and imaged using a confocal microscope (Nikon A1R). A negative control stain was also analyzed (p11 no Ig), which were cultures that underwent exposure to 100ng/mL p11 and the subsequent immunofluorescence protocol without inclusion of the anti-His tag primary antibody. The middle of the coverslip was found, and ten images across three technical replicates were taken at 20x magnification per treatment, with one image per replicate taken for the negative control (p11 no Ig).

p11 ELISA of isolated DRG neurons: Sensory neuron somata were cultured as mentioned in the Methods section. Cells were un-adhered with 0.05% trypsin, lysed with RIPA Lysis and Extraction Buffer (ThermoFisher, 89900), and underwent protein quantification as per attached instructions in the ELISA kit (Rat S100 Calcium Binding Protein A10 (S100A10) ELISA Kit, Biomatik, Cat# EKN48271-96T). Protein p11 was compared to the total protein of the lysate, quantified using bicinchoninic acid assay (BCA assay, Thermo Scientific, 23227).

Calcium imaging:

Schwann cells: Schwann cells were cultured in serum-containing media and plated onto glass coverslips. All reagents were obtained from ThermoFisher unless otherwise indicated. After two days in culture, coverslips were washed with extracellular buffer solution (150 mM NaCl, 10 mM HEPES, 8 mM glucose, 5.6 mM KCl, 2 mM CaCl2, and 1 mM MgCl2, pH 7.40 ± 0.03, and 320 ± 3 mOsm) for 30 minutes, incubated with 2.5 mg/mL Fura-

2-AM (Life Technologies) then washed for 30 minutes. Experimenters were blinded to genotype, and coverslips were mounted onto a perfusion chamber, placed on an inverted fluorescent microscope, superfused with extracellular buffer at 6 mL/min, and imaged at 20x magnification. Under light microscopy, the experimenter marked 20-50 Schwann cell bodies per coverslip prior to imaging; all marked Schwann cells were included in final analysis. Fluorescence images were captured at 340 nm and 380 nm with a cooled Andor Zyla sCMOS camera (Oxford Instruments) to calculate the bound to unbound ratio (340/380). NIS Elements software (Nikon) was used to detect and analyze intracellular calcium changes. Differing concentrations of ATP (1, 10, 50 μM) were applied for 10 seconds to the bath. Schwann cells that exhibited a >20% 340/380 response magnitude compared to baseline within 30 s after ATP application were considered responders.

Mass spectrometry:

Enzymatic "In Liquid" Digestion: Acellular rat cell culture media (~4-5ml) were speed-vac to ~250μl each, diluted 1:1 with water and proteins were extracted via TCA/Acetone precipitation for 45 minutes on ice to (10% TCA in 50% Acetone final vol:vol). Spun for 10 minutes at room temperature with max speed (16,000xg) and generated pellets were washed twice with cold (-20ºC) acetone. Generated protein pellets were solubilized and denatured in 15μl of 8M Urea in 50mM NH4HCO3 (pH 8.5). Subsequently diluted to 60μl for reduction step with: 2.5μl of 25mM Dithiothreitol (DTT) and 42.5μl of 25mM NH4HCO3 (pH 8.5). Incubated at 52°C for 15 minutes, cooled on ice to room temperature then 3μl of 55mM Chloroacetamide (CAA) was added for alkylation and incubated in darkness at room temperature for 15 minutes. Reaction was quenched by adding 8μl of 25mM DTT. Finally, 8μl of Trypsin/LysC solution (100ng/μl 1:1 Trypsin (Promega) and LysC (FujiFilm) mix in 25mM NH4HCO3) and 21μl of 25mM NH4HCO3 (pH8.5) was added to 100μl final volume. Digestion was conducted overnight at 37°C. Reaction was terminated by acidification with 2.5% Trifluoroacetic acid (TFA) to 0.3% final.

NanoLC-MS/MS: Peptides were analyzed by nanoLC-MS/MS using the Agilent 1100 nanoflow system (Agilent) connected to hybrid linear ion trap-orbitrap mass spectrometer (LTQ-Orbitrap Elite™ (ThermoFisher)) equipped with an EASY-Spray™ electrospray source (held at constant 35°C). Chromatography of peptides prior to mass spectral analysis was accomplished using capillary emitter column (PepMap® C18, 3μM, 100Å, 150x0.075mm (ThermoFisher)) onto which 2μl of extracted peptides was automatically loaded. NanoHPLC system delivered solvents A: 0.1% (v/v) formic acid , and B: 99.9% (v/v) acetonitrile, 0.1% (v/v) formic acid at 0.50 μL/min to load the peptides (over a 30 minute period) and 0.3μl/min to elute peptides directly into the nano-electrospray with

gradual gradient from 0% (v/v) B to 30% (v/v) B over 80 minutes followed by 5 minute fast gradient from 30% (v/v) B to 50% (v/v) B and concluded with a 5 minute flash-out from 50-95% (v/v) B. As peptides eluted from the HPLC-column/electrospray source survey MS scans were acquired in the Orbitrap with a resolution of 120,000 followed by CID-type MS/MS fragmentation of 30 most intense peptides detected in the MS1 scan from 350 to 1800 m/z; redundancy was limited by dynamic exclusion.

Data analysis: Elite acquired MS/MS data files were converted to mgf file format using MSConvert (ProteoWizard: Open-Source Software for Rapid Proteomics Tools Development). Resulting mgf files were used to search against Uniprot Rattus norvegicus proteome database (UP000002494, 10/06/2020 download, 31,681 total entries) along with a cRAP common lab contaminant database (116 total entries) using in-house Mascot search engine 2.7.0 (Matrix Science) with fixed Cysteine carbamidomethylation and variable Methionine oxidation plus Asparagine or Glutamine deamidation. Peptide mass tolerance was set at 10 ppm and fragment mass at 0.6 Da. Protein annotations, significance of identification and spectral based quantification was done with Scaffold software (version 4.11.0, Proteome Software Inc.). Peptide identifications were accepted if they could be established at greater than 88.0% probability to achieve an FDR less than 1.0% by the Scaffold Local FDR algorithm. Protein identifications were accepted if they could be established at greater than 6% probability to achieve an FDR less than 1.0% and contained at least 2 identified peptides. Protein probabilities were assigned by the Protein Prophet algorithm [5] that contained similar peptides and could not be differentiated based on MS/MS analysis alone were grouped to satisfy the principles of parsimony. Proteins sharing significant peptide evidence were grouped into cluster.

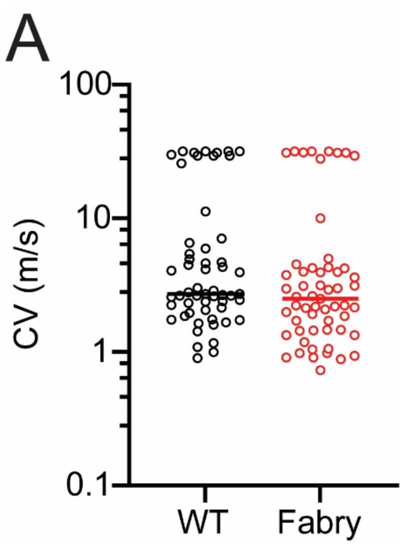

<u>Figure S1: Fibers from Fabry rats exhibit similar conduction velocities compared to WT.</u> A) Conduction velocity values of all fibers recorded for evoked response firing frequency from Fabry rats compared to fibers recorded from WT rats. n = 55-57 fibers from 3 animals per genotype. Values reported as median. unpaired *t*-test. CV = conduction velocity.

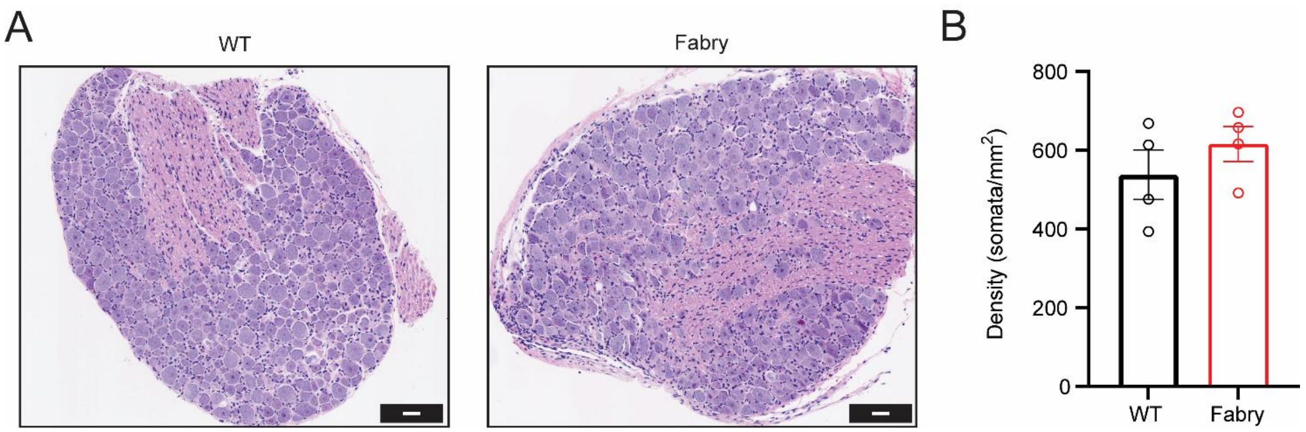

<u>Figure S2: Lumbar DRG isolated from Fabry and WT rats exhibit similar neuron soma density.</u> A) Representative cross-sections of lumber (L1) DRG from WT and Fabry rats, stained with hematoxylin and eosin (H&E), scale bar 50μm. B) Both genotypes exhibit a similar density of somata per DRG, Values reported as mean ± SEM. (B) unpaired *t*-test. n = 4 animals per genotype, one DRG cross section analyzed per animal.

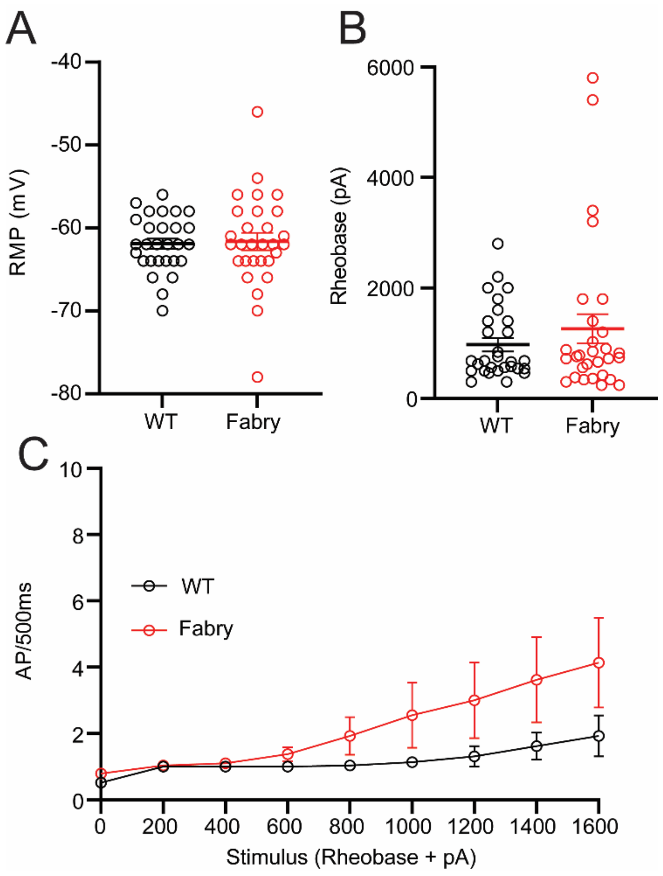

Figure S3: Large diameter DRG neuron somata isolated from Fabry and WT rats exhibit similar current-evoked excitability. A) Both Fabry and WT large diameter (>32 μm) exhibit similar resting membrane potentials and B) rheobase. C) There was a statistically insignificant trend toward increased firing frequency to suprathreshold current stimulation starting from rheobase to rheobase + 1,600 pA in Fabry DRG neuron somata. n = 29 neurons from 8 animals per genotype. Values reported as mean ± SEM. (A, B) unpaired *t*-test, (C) two-way repeated measures ANOVA, main effect of genotype, $p = 0.1196$. interaction between genotype and stimulus, $p = 0.077$. RMP = resting membrane potential, AP = action potential.

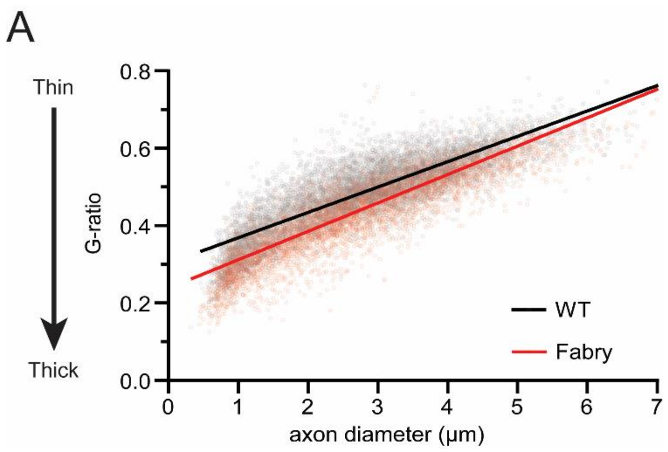

Figure S4: Fabry and WT axon G-ratio derived from tibial nerve fascicles plotted against axon diameter. Simple linear regression of axon g-ratios plotted against axon diameter from cross-sectioned tibial nerves of WT and Fabry rats. n = g-ratio from 7,000 – 8,000 axons derived from cross-sectioned tibial nerves of 6 animals per genotype.

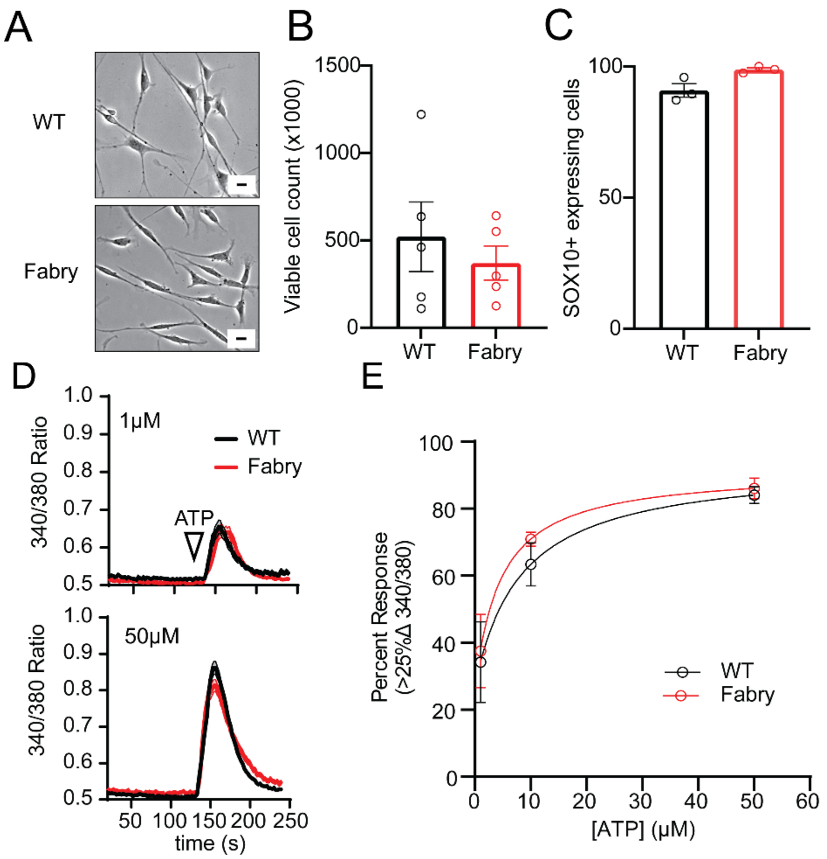

Figure S5: Fabry and WT Schwann cells exhibit similar cell health. A) Representative light microscopy images of Fabry and WT Schwann cells in culture, scale bar 10 μm. B) WT and Fabry Schwann cell cultures possessed similar cell viability via Trypan blue analysis two days after plating. C) Cultured Schwann cells from both genotypes exhibited high purity two days after plating as quantified by immunofluorescent imaging with the Schwann cell marker SOX10 (each dot represents the averaged SOX10+ cell percentage gathered from 3 animals per genotype). D) Aggregated traces depicting the mean ± SEM of WT and Fabry Schwann cells exposed to 1 or 50 μM ATP for 10s. E) Calcium imaging analysis revealed a similar concentration-dependent response to ATP in both genotypes. (B, C) each dot represents per animal measurement. (D, E) n = 200 Schwann cells per group from 3 animals. Values reported as mean ± SEM.

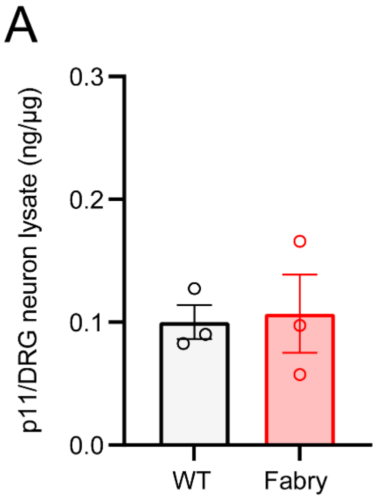

Figure S6: Expression of protein p11 is similar between Fabry and WT DRG isolated DRG neuron somata. A) Amount of protein p11 from cellular lysates of cultured DRG neuron somata compared to total protein concentration. n = culture lysates from 3 animals per genotype. unpaired *t*-test.

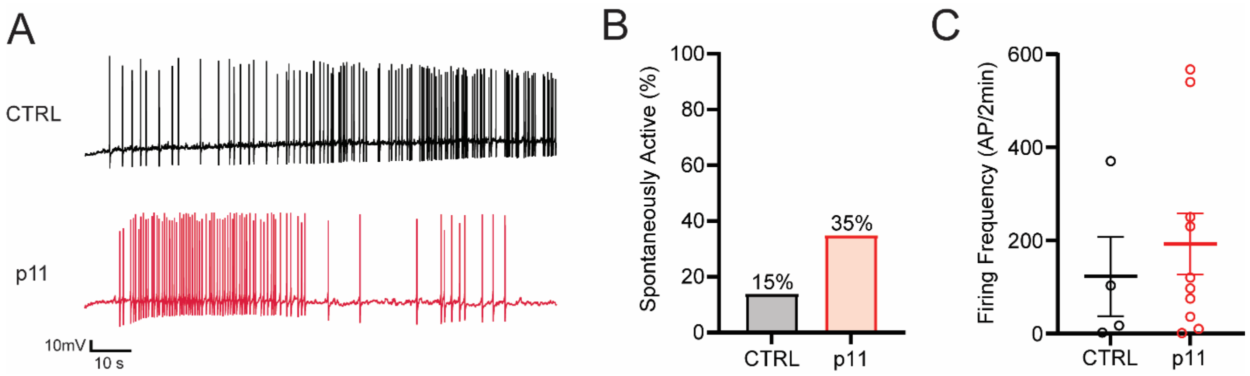

<u>Figure S7: Naïve neurons exposed to overnight incubation of p11 exhibit a non-significant trend towards increased spontaneous activity.</u> A) Representative spontaneous traces of naïve DRG neurons exposed to 100 ng/mL of p11 or CTRL. B) A trending, but not significant, number of neurons exposed to p11 exhibited spontaneous activity compared to CTRL neurons. n = 28 neurons per treatment from 7 animals C) Spontaneous firing frequency of DRG neurons were similar between treatments. (B) reported as mean, (C) reported as mean ± SEM. (B) χ^2, p = 0.12, (C) unpaired *t*-test.

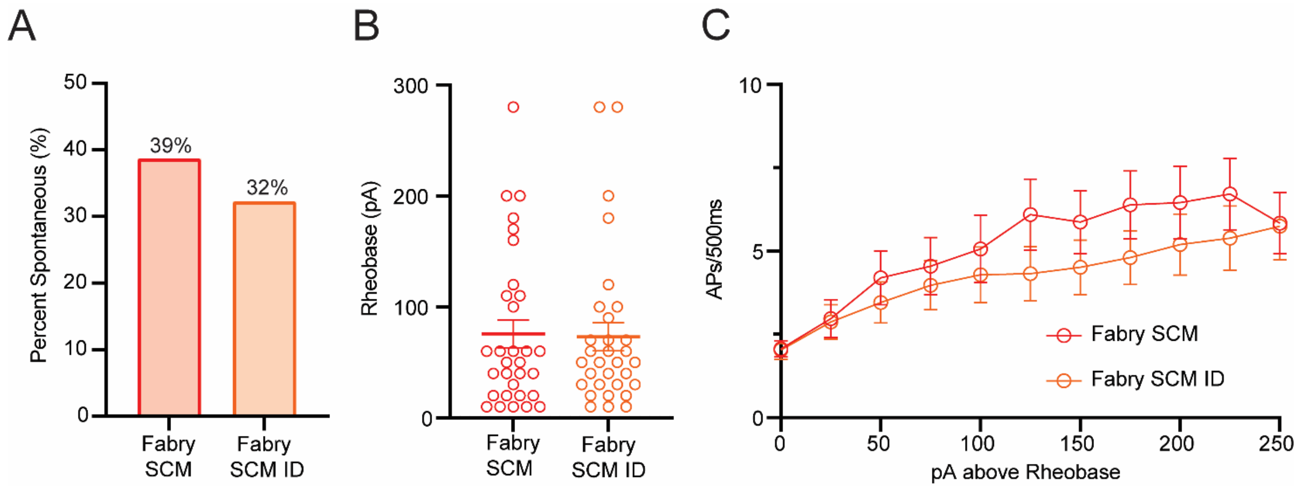

Figure S8: Naïve neurons treated with either Fabry SCM or Fabry SCM ID exhibit similar spontaneous activity and current-evoked excitability. A) A similar proportion of DRG neuron somata exhibit spontaneous activity when exposed to Fabry SCM or Fabry SCM with immunodepleted p11 (ID) overnight. B) DRG neurons exposed to either treatment exhibit a similar rheobase and C) current-evoked firing frequency. n = 31 neurons per treatment from 8 animals. (A) reported as mean and (B) mean ± SEM. (A) χ^2, (B) unpaired *t*-test, (C) two-way repeated measures ANOVA. Fabry SCM = Fabry Schwann cell conditioned media. Fabry SCM ID = Fabry Schwann cell conditioned media with immunodepleted p11.

Small Diameter (≤ 32um)		
	**WT**	**Fabry**
Cell Number	72	70
RMP (mV)	-57.7 ± 0.9	-56.9 ± 1.1
**Spontaneous Activity**		
Cell Number	34	33
Cell Size (µm)	24.02 ± 0.4	24.30 ± 0.6
Capacitance (pF)	43.75 ± 2.9	37.6 ± 2.5
Percent Spontaneous (%) [b]	11.7%	33.3% *
Firing Frequency (AP/2min)	424.3 ± 289.9	201.5 ± 80.92
**Current-Evoked Excitability**		
Cell Number	38	37
Cell Size (µm)	25.3 ± 0.5	25.07 ± 0.6
Capacitance (pF)	37.7 ± 1.9	37.9 ± 2.3
Input Resistance (mΩ)	561.3 ± 48.9	487.2 ± 57.6
Rheobase (pA) [a]	300.0 ± 39.1	202.0 ± 22.8 *
AP Threshold (mV)	-19.4 ± 1.6	-21.5 ± 1.4
AP Amplitude (mV)	42.3 ± 1.6	42.0 ± 1.8
AP Half-width (ms)	1.6 ± 0.09	1.5 ± 0.09

Table 1: Passive and current-evoked membrane properties of small diameter DRG neurons isolated from WT or Fabry rats. Unpaired *t*-test = [a], * $p < 0.05$. χ^2 main effect of treatment = [b], Fisher's exact post hoc comparisons, * $p < 0.05$. RMP: resting membrane potential, AP: action potential. Reported as Mean ± SEM.

Large Diameter (> 32um)		
**Current-Evoked Excitability**		
	**WT**	**Fabry**
Cell Number	29	29
Cell Size (μm) [a]	39.1 ± 1.0	43.9 ± 1.7 *
Capacitance (pF)	92.12 ± 9.0	103.9 ± 10.5
RMP (mV)	-61.9 ± 0.6	-61.6 ± 1.1
Input Resistance (mΩ)	127.2 ± 18.3	185.0 ± 30.8
Rheobase (pA)	976.6 ± 121.6	1264.0 ± 264.9
AP Threshold (mV)	-28.8 ± 2.0	-26.2 ± 2.7
AP Amplitude (mV)	47.2 ± 2.1	47.4 ± 1.7
AP Half-width (ms)	0.93 ± 0.09	1.16 ± 0.19

Table 2: Passive and current-evoked membrane properties of large diameter DRG neurons isolated from WT or Fabry rats. Unpaired *t*-test = [a], * $p < 0.05$. RMP: resting membrane potential, AP: action potential. Reported as Mean ± SEM.

	CTRL SCM	WT SCM	Fabry SCM
Cell Number	51	59	60
RMP (mV) [a]	-56.1 ± 1.1	-54.8 ± 1.1	-51.0 ± 1.1 ** #
**Spontaneous Activity**			
Cell Number	23	28	29
Cell Size (μm)	25.5 ± 0.3	24.2 ± 0.4	24.30 ± 0.8
Capacitance (pF)	34.8 ± 2.1	33.4 ± 2.8	28.3 ± 2.5
Percent Spontaneous (%) [b]	4%	18%	52% ** #
Firing Frequency (AP/2min)	5 ± 0	484 ± 149.3	295.8 ± 137.2
**Current-Evoked Excitability**			
Cell Number	28	31	31
Cell Size (μm)	25.4 ± 0.3	24.6 ± 0.6	24.2 ± 0.5
Capacitance (pF)	34.0 ± 2.8	33.2 ± 2.8	25.9 ± 2.1
Input Resistance (mΩ)	411.4 ± 39.5	501.3 ± 49.4	615.9 ± 82.1
Rheobase (pA)	291.4 ± 87.7	206.1 ± 23.8	133.2 ± 18.0
AP Threshold (mV)	-21.2 ± 1.7	-22.8 ± 1.3	-19.7 ± 1.7
AP Amplitude (mV)	43.9 ± 1.3	42.1 ± 2.1	41.8 ± 1.9
AP Half-width (ms)	1.67 ± 0.1	1.66 ± 0.09	1.61 ± 0.09

Table 3. Membrane properties of DRG neurons isolated from naïve rats following overnight treatment of WT, Fabry, or unconditioned (CTRL) Schwann cell media (SCM). One-way ANOVA main effect of treatment = [a] ; Bonferroni post-hoc tests. χ^2 main effect of treatment = [b] ; Fisher's exact post hoc comparisons. CTRL vs. Fabry = *, ** $p < 0.01$, CTRL vs. Fabry #, # $p < 0.05$). RMP: resting membrane potential, AP: action potential. Reported as Mean ± SEM.

Upregulated Protein Release (Fabry)			
**Protein**	**WT (Normalized Spectra)**	**Fabry (Normalized Spectra)**	**Fold Change (Fabry)**
p11 (S100A10)	0.0 ± 0.0	0.93 ± 0.27 **	∞
GSDMA	0.0 ± 0.0	0.94 ± 0.31 *	∞
TGM1	0.14 ± 0.14	1.69 ± 0.57 *	11
TPI1	0.63 ± 0.27	1.53 ± 0.25 *	2.4
**Downregulated Protein Release (Fabry)**			
**Protein**	**WT (Normalized Spectra)**	**Fabry (Normalized Spectra)**	**Fold Change (Fabry)**
ENO2	3.59 ± 0.16	1.69 ± 0.33 ***	0.5
PKM	14.13 ± 1.29	7.21 ± 1.50 ***	0.5
GPI	7.18 ± 1.04	3.93 ± 0.58 *	0.5
TAGLN2	2.12 ± 0.46	0.76 ± 0.20 *	0.5
EEF2	10.38 ± 1.54	5.51 ± 1.11 *	0.4
PSMA6	1.16 ± 0.35	0.21 ± 0.21 *	0.5
EIF5A	0.85 ± 0.36	0.0 ± 0.0 *	0

Table 4. Significantly different protein concentrations between WT and Fabry Schwann cell media as identified by NanoLC-MS/MS analysis. Benjamini-Hochberg corrected two-tailed *t*-test, * $p < 0.05$, ** $p < 0.01$, *** $p < 0.001$. Reported as Mean ± SEM.

	CTRL	p11
Cell Number (total)	28	28
Cell Size (μm)	26.7 ± 0.4	25.3 ± 0.4
Capacitance (pF)	35.4 ± 2.1	31.7 ± 1.6
RMP (mV)	-58.6 ± 1.4	-52.1 ± 1.6 **
**Spontaneous Activity**		
Percent Spontaneous (%)	14%	35%
Firing Frequency (AP/2min)	123.0 ± 85.3	207.6 ± 65.7
**Current-Evoked Excitability**		
Input Resistance (mΩ)	466.3 ± 38.8	535.7 ± 40.1
Rheobase (pA)	174.6 ± 25.8	92.1 ± 22.8 *
AP Threshold (mV)	-15.4 ± 1.7	-15.1 ± 1.9
AP Amplitude (mV)	43.5 ± 1.2	41.2 ± 1.3
AP Half-width (ms)	1.94 ± 0.15	1.90 ± 0.11

Table 5. Passive and current-evoked membrane properties of DRG neurons isolated from naïve rats following overnight treatment of 100ng/mL p11 or vehicle (CTRL). Unpaired *t*-test, * $p < 0.05$, ** $p < 0.01$. RMP: resting membrane potential, AP: action potential. Reported as Mean ± SEM.

	Fabry SCM	Fabry SCM ID
Cell Number (total)	31	31
Cell Size (μm)	23.2 ± 0.3	23.5 ± 0.4
Capacitance (pF)	24.0 ± 1.0	25.8 ± 1.2
RMP (mV)	-49.1 ± 1.3	-54.6 ± 1.5 **
**Spontaneous Activity**		
Percent Spontaneous (%)	38.7%	32.3%
Firing Frequency (AP/2min)	222.8 ± 97.3	49.4 ± 33.2
**Current-Evoked Excitability**		
Input Resistance (mΩ)	817.6 ± 91.0	620.1 ± 41.8
Rheobase (pA)	75.81 ± 12.6	73.2 ± 12.7
AP Threshold (mV)	-16.9 ± 1.9	-17.8 ± 1.8
AP Amplitude (mV)	39.8 ± 2.1	43.0 ± 2.1
AP Half-width (ms)	1.9 ± 0.09	1.8 ± 0.09

Table 6: Passive and current-evoked membrane properties of DRG neurons isolated from naïve rats following overnight treatment of Fabry Schwann cell media (SCM) or Fabry SCM with immunodepleted (ID) p11. Unpaired *t*-test, ** $p < 0.01$. RMP: resting membrane potential, AP: action potential. Reported as Mean ± SEM.

Supplemental References:

1. Waltz, T.B., et al., *Sensory-specific peripheral nerve pathology in a rat model of Fabry disease.* Neurobiol Pain, 2021. **10**: p. 100074.
2. Lewis, T.R., et al., *Cos2/Kif7 and Osm-3/Kif17 regulate onset of outer segment development in zebrafish photoreceptors through distinct mechanisms.* Developmental biology, 2017. **425**(2): p. 176-190.
3. Sadler, K.E., et al., *Peripheral nerve pathology in sickle cell disease mice.* Pain reports, 2019. **4**(4): p. e765-e765.
4. Marshall, A. and L.W. Duchen, *Sensory system involvement in infantile spinal muscular atrophy.* Journal of the Neurological Sciences, 1975. **26**(3): p. 349-359.
5. Nesvizhskii, A.I., et al., *A statistical model for identifying proteins by tandem mass spectrometry.* Anal Chem, 2003. **75**(17): p. 4646-58.
